# Supplementary material for: Genome-Wide Association Studies for Methane Production in Dairy Cattle
Source: Genes (Basel). 2019 Dec 2;10(12):995. doi: 10.3390/genes10120995 (PMC6969927; doi:10.3390/genes10120995)
Supplement: Supplementary file 1 [file genes-10-00995-s001.zip › Suplementary files/Figure S1.pdf]

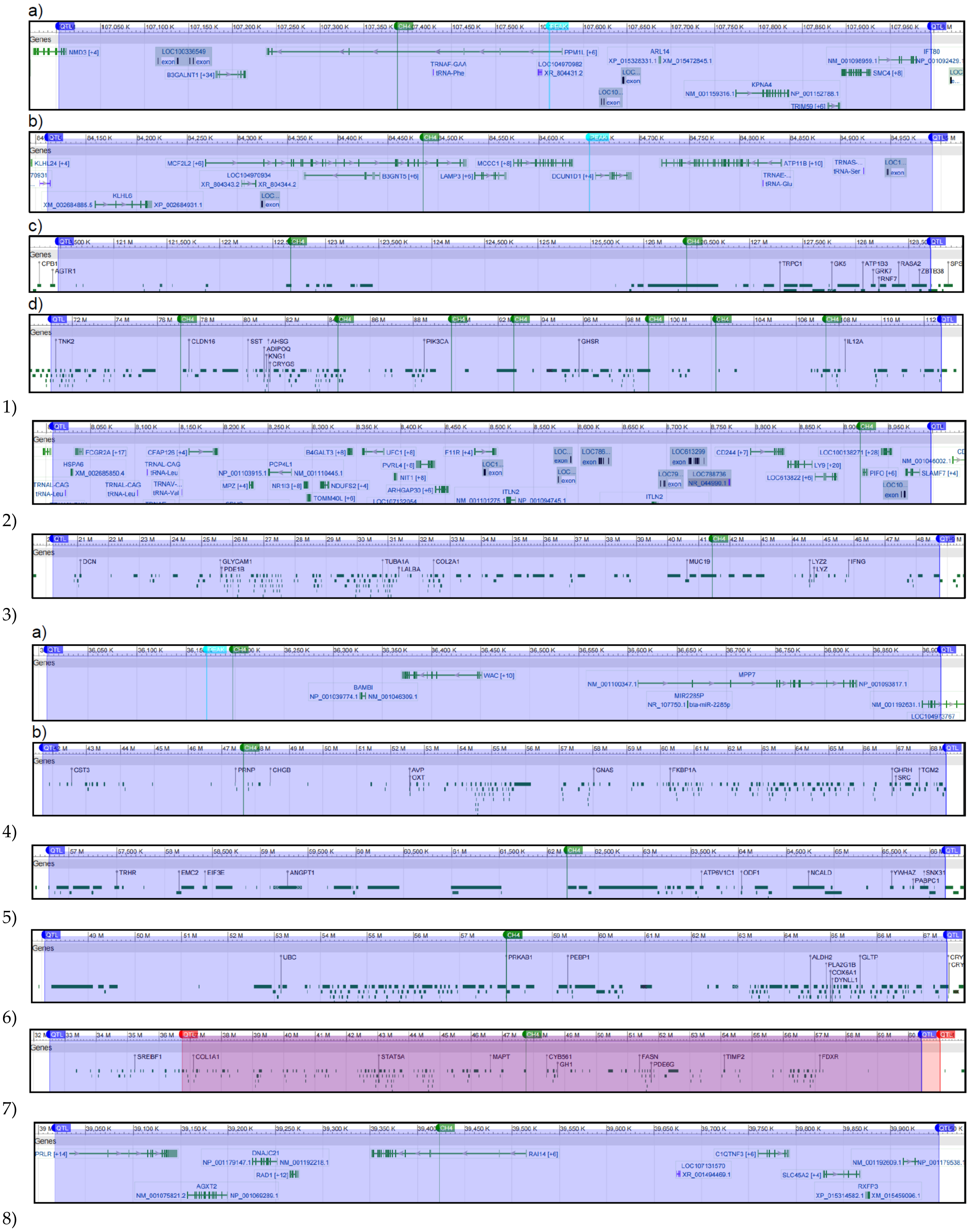

Figure S1. QTLs and genes associated with methane production during milking (MEIm). Blue and red flags represent the QTLs, green flags represent the SNPs associated with MEIm and the green lines represent the genes. 1a) a QTL related to dry matter intake was found, and it can be seen that the QTL peak and the MEIm marker are in the same *PPM1L* gene. 1b) a marker associated with MEIm within a QTL linked to the trans-6/9-C18 fatty acid:1, this marker is found in the *MCF2L2* gene and is very close to the QTL peak. 1c) two markers associated with MEIm were located within the QTL associated with the percentage of myristic acid in milk, one in the *SLC9A9* gene and the other in the *LOC104971015* gene. 1d) markers associated with MEIm inside the QTL for daily weight gain; this is a relatively broad QTL, within which we found 7 markers associated with MEIm, going through the genes *LOC107132190*, *LOC104970931*, *LOC100138913*, *NAALADL2*, *MECOM*, and *PPM1L*. 2) a marker associated with MEIm that is found inside the QTL associated with daily weight gain. 3) a marker associated with MEIm within the QTL for intramuscular fat, on the *SLC2A13* gene. 4a) a marker associated with MEIm that is in the QTL related to the content of the fatty acids C22:1 and trans-12-C18:1, this is very close to the QTL peak. 4b) a marker associated with MEIm was found within the QTL associated with the percentage of caproic acid in milk, on the *SLC23A2* gene. 5) a marker associated with MEIm was found within the QTL associated with the percentage of palmitoleic acid in milk. 6) two markers associated with CH_4 were found within the QTL associated with fatty acids in milk, both on the *TMEM233* gene. 7) a marker associated with CH_4 was found within two QTL associated with fatty acids in milk, on the *MRC2* gene. 8) a QTL associated with fatty acids in milk that has a marker associated with CH_4 on the *RAI14* gene.
