# Supplementary material for: Genome-Wide Association Studies for Methane Production in Dairy Cattle
Source: Genes (Basel). 2019 Dec 2;10(12):995. doi: 10.3390/genes10120995 (PMC6969927; doi:10.3390/genes10120995)
Supplement: Supplementary file 1 [file genes-10-00995-s001.zip › Suplementary files/Table S1.pdf]

Supplementary Table 1. SNPs associated to methane production in this study.

Marker	Chromosome	Position	Regression -log10 P	MAF
BovineHD0100039506	1	138388705	5.273557859	0.05197
BovineHD0100033007	1	116746240	4.874903676	0.05357
BovineHD0500011829	5	41431327	4.690852735	0.05535
BovineHD0100026381	1	92730269	4.401806765	0.09286
BovineHD0100035695	1	126404986	4.381196517	0.05536
BovineHD0900021600	9	77448787	4.35370731	0.06607
BovineHD1400017260	14	62204044	4.328259396	0.05357
BovineHD0100024203	1	84485319	4.064959741	0.05893
BovineHD0300026630	3	92487386	3.960462062	0.05000
Hapmap48751-BTA-41232	17	58016927	3.796795488	0.36964
ARS-BFGL-NGS-109467	13	28331553	3.780437974	0.41756
BovineHD1700016427	17	58018395	3.61682694	0.37950
BovineHD0100022218	1	77090546	3.570940261	0.05714
BovineHD1100001194	11	3312404	3.554029708	0.27061
BovineHD0400033637	4	115960201	3.547520868	0.05893
ARS-BFGL-NGS-12759	28	15739975	3.543310713	0.46250
BovineHD0100025559	1	89815855	3.541435653	0.08750
BovineHD1300013919	13	47642260	3.53034743	0.07500
BovineHD2300000102	23	732008	3.502963271	0.08214
BovineHD1700001005	17	4159032	3.479003084	0.31964
BovineHD0300029008	3	101384522	3.456878439	0.05000
BovineHD0100029171	1	102217070	3.432251694	0.08571
BovineHD2000003504	20	11023816	3.391793093	0.06964
BovineHD1300010064	13	34736213	3.374928078	0.06318
ARS-BFGL-BAC-34879	20	39422828	3.34872604	0.33571
Hapmap45550-BTA-32092	13	36197476	3.346154758	0.38710
BovineHD1500026056	15	83740340	3.343468023	0.07500
BovineHD0400035603	4	16265225	3.326690896	0.05000
BovineHD0100028269	1	99062029	3.318280412	0.08214
BovineHD2000001637	20	5189875	3.281553774	0.41607
ARS-BFGL-NGS-25983	19	47747001	3.271864676	0.12734
BTA-121571-no-rs	3	3634590	3.214016868	0.17143
BovineHD2000002522	20	7901522	3.180174107	0.10357
BovineHD0300023978	3	83877749	3.173221479	0.05000
Hapmap27254-BTA-49411	1	122672541	3.168963439	0.07857
BovineHD0100030410	1	107388026	3.153257752	0.07679
BTA-112161-no-rs	13	22904772	3.119304518	0.36786
Hapmap50820-BTA-91209	2	35993314	3.1122061	0.47679
BTB-00493094			3.111939742	0.38214
BovineHD0300010316	3	33082130	3.103122015	0.42832
BovineHD0300002944	3	8918173	3.064355408	0.47122
BovineHD1400002085	14	7744264	3.047997381	0.08065
BovineHD0400033369	4	115211711	3.024311604	0.26344

BovineHD0300023691	3	82504149	3.022452258	0.28036
BovineHD1600021933	16	76168239	3.019162345	0.18638
BovineHD2300001082	23	4709756	3.011305913	0.16045
